# How to Control the Distribution of Anchored, Mn_12_–Stearate, Single-Molecule Magnets

**DOI:** 10.3390/nano9121730

**Published:** 2019-12-04

**Authors:** Magdalena Laskowska, Oleksandr Pastukh, Dominika Kuźma, Łukasz Laskowski

**Affiliations:** Institute of Nuclear Physics Polish Academy of Sciences, PL-31342 Krakow, Poland; magdalena.laskowska@ifj.edu.pl (M.L.); oleksandr.pastukh@ifj.edu.pl (O.P.); dominika.kuzma@ifj.edu.pl (D.K.)

**Keywords:** single-molecule magnet, Mn12, surface functionalization, separation

## Abstract

Controlling the distribution of the Mn_12_–stearate, single-molecule magnets (SMMs) anchored on a select surface is expected to be a new method for tuning its interactions, and an investigation on the magnetic properties of separated magnetic molecules is also lacking. The anchoring of the SMMs at the surface with an assumed statistic distance between each other is not an easy task; nevertheless, in this work, we show a synthesis which allows for this in detail. The immobilization of the Mn_12_–stearate was demonstrated with the use of FTO glasses and spherical silica as substrates. Based on differential pulse anodic stripping voltammetry (DPASV) and transmission electron microscopy (TEM) observations, we proved the efficiency of the method proposed. We observed continuous decreasing the number of bonds, and afterward, decreasing in the number of immobilized molecules with an increasing the number of spacer units used for separation of the magnetic particles.

The current developments in surface chemistry provide excellent opportunities for creating new materials and exploring novel and unique phenomena that take place on the surfaces of substances. In that vein, the functionalization of a surface plays an important role, and there are a great variety of techniques and methods aimed at modification of surfaces of nanoscale structures [1]. Surfaces of solids such as silica, glass, or carbon provide the opportunity to create unusual arrangements of atoms and molecules and also to connect materials with entirely different properties [2]. On the other hand, since every chemical modification changes the functionality of a surface, some significant effects, in contrast to “bulk” properties, should be taking into account [3].

For the organic functionalization of semiconductors silicon surfaces, self-assembled monolayers and direct attachment of oxygen- and carbon-free chemical functions [4] can be used. Additionally, introducing organic functional groups on silica surfaces is possible by the use of the immobilization of a monomeric [5] or polymeric [6] silane precursor. Also, a new class of materials, which is attractive from the functionalization point of view and characterized by large specific surface areas, is silica-based mesoporous materials. The incorporation of some functional units, in this case, can be achieved by applying grafting or co-condensation methods or using periodic mesoporous organosilicates [7]. Such a material has potential applications in the fields of catalysis and environmental remediation [8], or for use as a tunable, nonlinear optical material [9] for the performance of optoelectronic devices.

A glass surface can also be used as a substrate to produce functional materials. In this case, using zeolite microcrystals as model microbuilding blocks [10] opens up a possible application of zeolites as advanced materials. A more promising material for surface modification is Fluoride doped Tin Oxide (FTO) coated glass, which shows efficient surface chemical functionalization through electrochemical grafting [11] or by electrostatic self-assembled monolayers of transition metal cations receptors [12].

However, an essential issue of surface functionalization, which was not included in aforementioned techniques, is the possibility to separate the individual functionalities and to control not only distances between them but their interactions. Such a solution is possible to realize using spacer units [13], which gives the possibility of horizontal distributions of the functional groups. Such an approach is a critical factor of intermolecular interaction controlling and opens up extensive opportunities for practical applications. An interesting and promising option is deposition on the surface of magnetic molecules [14]. As it was shown in [15], attaching Mn_12_–stearate single-molecule magnets (SMMs) to the surface of spherical silica, allows one to separate the individual molecular magnets.

Here we present a procedure that provides a possibility of controlling statistic distance between SMMs on a surface by using spacer units. Theoretically, it is possible not only to separate the SMMs but also to tune mean intermolecular distances between SMMs by adjusting the number of spacer molecules: more separator molecules with regard to anchoring units, means a larger distance between anchored functionalities. In this study, we experimentally demonstrate this idea for the case of SMMs. We propose anchoring the Mn_12_–stearate single-molecule magnets onto a surface of two types of substrates. The first one is FTO covered glass. The conductive substrate allows for the investigation of the samples utilizing electrochemistry methods. The second one is spherical nanosilica, allowing for the direct observation the Mn_12_-stearate SMMs [15]. As the anchoring units we applied propyl carbonic acid groups, while the role of spacers was played by trimethoxysilane. The schema of the material and illustration of proposed idea is shown in Figure 1.

The concept is a combination of those described earlier by our team [13,15]. After preliminary testing, the procedure worked predictably, according to our assumption, at first glance. After careful analysis and comparison of the results of electrochemical measurements and TEM observation, nevertheless, it turned out that the Mn_12_–stearate immobilization mechanism was more complicated than we assumed. The finding presented in this work seems to be very important for controlling the distribution of single-molecule magnets on the surface. The procedure allows for obtaining separated SMM on the surface and tuning the statistic intermolecular distances. The ability to attach isolated magnetic molecules on the surface will enable us to directly investigate their properties as single molecules in contrast to their study or extrapolation of their properties using a bulk analysis. The use of this approach opens a wide possibility for synthesis of materials that can be used in molecular-based magnetic devices (such as data storage and spintronics devices) and in quantum computation.

As mentioned before, we prepared samples in two forms: based on FTO-glass (1), and as a fine powder based on spherical silica (2). Both were prepared in an analogous way. The synthesis routes involve a few steps, as illustrated in Figure 2.

Before the proper chemical processing, we prepared substrates: FTO glasses (3D Nano Ltd., Krakow, Poland) were degreased by the use of mechanical washing with a regular detergent. Next, the substrates were soaked in a solution containing a mixture of concentrated HCl (Poch Inc., Gliwice, Poland) and methanol (1:1 of the volume) for one hour. After this process, we washed the substrates with distilled water and dried them by flowing dry nitrogen. Spherical silica was synthesized as described in the literature [16], and used as prepared. Samples based FTO-glass were named *FTO-COOMn*_12_, while those based on spherical silica hereafter are called *SILS-COOMn*_12_. The synthesis of Mn_12_–stearate was performed according to the protocol described previously [17,18].

In Step I we grafted the substrates with the precursors of the anchoring units and spacer groups. To that end, the (***Solution I***) was prepared. It contained the mixture of butyronitrile triethoxysilane (BNTES—Sigma-Aldrich, Saint Louis, MI, USA) and tetraethyl orthosilicate (TEOS—Sigma-Aldrich Inc., Saint Louis, MI, USA) in toluene. The total concentration of TEOS and BNTES was 10^−2^ mol/dm^3^ for both substrates, while their molar proportion determined the distances between functional units: BNTES created the anchoring group for the functionality, while TEOS was a precursor of the spacer unit. We applied various proportions of BNTES and TEOS—1:0, 1:3, 1:6, 1:9, 1:12, and 1:15—which corresponded to the number of spacer units for each one anchoring molecule. In order to distinguish the samples, we added the number of spacers after the name; for example *SILS-COOMn*_12_*N*0 means spherical silica-based material does not contain any separators (N0—only BNTES), while sample *SILS-COOMn*_12_*N*15 includes 15 spacers per one anchoring unit (N15—initial proportion BNTES:TEOS, 1:15 molar ratio). Analogously, we had *FTO-COOMn*_12_*N*0 with no spacers and *FTO-COOMn*_12_*N*15 with 15 spacers.

For the case of ***FTO coated glass*** we immersed them into ***Solution I*** for 24 h. Subsequently, we washed the pre-functionalized glasses by immersing in the toluene ultrasonic bath for 1 hour in order to remove any excess of silane from the surface and to avoid the polymerization. Next, we rewashed the substrates and dried them. As a result, we obtained FTO glasses which contained cyanopropyl and hydroxyl units on the surfaced in concentrations depending on the initial molar proportions of BNTES and TEOS.

In the Step II we silylized our materials: hydroxyl units were converted into trimethyl silane groups (constituting spacers). To do this we treated samples with a solution of chlorotrimethylsilane (ClTMS) in toluene (2% of volume)—Solution II. Pre-functionalized FTO glasses were immersed in this solution and for 24 h at room temperature. After this process the samples were rinsed with toluene and acetone. After drying under vacuum for two hours samples were ready for further processing.

During the (***Step III***) the cyanopropyl units were transformed into propyl carbonic acid groups (constituting the anchors for Mn_12_-stearate molecules) by hydrolysis. To do this we soaked the samples in the concentrated hydrochloric acid (37%—***Solution III***) for at least 5 h. Next, we rinsed the samples several times with deionized water, and dried under vacuum for two hours.

In the last step, we functionalized the anchoring units (carbonic acid) by Mn_12_–stearate molecules (**Step IV**). To do this we immersed the substrates containing propyl carboxy units in the solution of Mn_12_–stearate in dichloromethane (0.1 g of Mn_12_–stearate in 50 mL of CH_2_Cl_2_—(***Solution IV***)). The procedure was carried out in a weighing dish and under ultrasonification for 20 h. After this we rinsed the samples by the solvent for a few times to remove residues of the doping agent, and dried them under vacuum for a few hours. The resulting material was meant to posses SMMs immobilized at the substrate’s surface via bounding by propyl carbonic acid anchoring units. Functionalized FTO glasses were stored in a refrigerator under an argon atmosphere.

Similar steps were applied for ***spherical silica***. Firstly, the spherical silica was mixed with ***Solution I*** under reflux overnight (0.5 g of spherical silica per 50 mL of the ***solution I***). Next, we recovered the product by centrifugation and washed it with pure toluene. We repeated this procedure four times, and subsequently dried the resulting powder under vacuum.

Next, the silylation was carried out by mixing the pre-functionalized powder with ***Solution II*** under reflux, similarly to what is described above.

Hydrolysis was carried out by concentrated hydrochloric acid. We performed the reaction under reflux (12 h). Subsequently, we centrifuged the resulting powder and washed it by water and acetone (four times, until neutral pH was reached).

Finally, we functionalized the silica containing carbonic acid anchoring units on the surface with Mn_12_–stearate. For the functionalization of 0.3 g of silica, we applied 50 mL of the ***Solution IV*** (for all concentrations of the anchoring units). We mixed this suspension overnight at room temperature under an argon protective atmosphere. The resulting powders (*SILS-COOMn*_12_*NX*, where X was a number of spacers per one anchoring unit) were recovered by centrifugation, washed by dichloromethane five times, and dried under vacuum for 10 h. Samples were stored in an argon atmosphere in a refrigerator.

We assumed that by the variation of the number of spacers, it would be possible to control the distribution of the anchored single-molecule magnets. To check our assumption, we decided to carry out two crucial investigations: differential pulse anodic stripping voltammetry (DPASV) for the case of samples prepared on FTO-glasses and TEM microscopy for spherical silica-based materials. The DPASV is a susceptible technique allowing for quantitative estimation of the number of the bonds between substrate and SMMs [19,20]. TEM microscopy (FEI Tecnai G2 20 X-TWIN with an LaB6 emission source and an FEI Eagle 2 K CCD camera - Thermo Fisher Scientific Inc., Waltham, MA, USA), in turn, allows for direct observation of the SMMs anchored at the surface of spherical silica, as we have shown before [15]. Measurements of the pristine substrates as a reference are covered in Supplementary Materials.

The DPASV spectra (SP200 potentiostat/galvanostat, BioLogic Inc., Seyssinet-Pariset, France) of the FTO-glass-based samples are shown in Figure 3. The spectra are quite noisy (normal taking into account that a relatively low amount of the detected specimen), but DPASV peaks are clearly visible. For all samples, we can see characteristic stripping asymmetric peaks at +0.28 V. This confirms the presence of the anchored molecules: Mn_12_ in this case.

The position of the DPASV peak suggested that we were dealing with chemically bonded specimens [19]. The relatively high value of the potential is typical for specimens bonded via covalent-polarized bonds of carbonic units [13,21], not only physically adsorbed ones. What is more, the peaks are well-localized. On this basis, we can conclude that a single type of ionization connected with charge transport, and, in our opinion, that was stripping bonds between carbonic groups and the SMMs. Another fact arguing for this, is that we did not adsorb any SMMs molecules at the supports not-containing anchoring units (see Supplementary Materials). Naturally, we need to take into account the possible decomposition of the Mn_12_–stearate molecules as well. In our opinion, however, this is unlikely, since in such a case, we would expect the peak or rather peaks’ positions to be different than what is typical for the stripping of carbonic bonds. Of course, we cannot exclude such a possibility.

The relative amounts of bounds can be estimated by measuring the area under the DPASV peaks [19] (see values given in Figure 3). The comparison of the relative amount of the bounds was according to our expectations at first glance. More spacer units caused less bonds between the surface and SMMs. We must emphasize nevertheless, that it does not mean less of the anchored magnetic molecules at all. To understand this, let us consider TEM microscopic images for the samples based on spherical silica with the concentration of the anchored single-molecule magnets corresponding to the materials based on FTO-glasses. The resulting images can be seen in Figure 4.

The TEM results are quite surprising. For all cases, one can see separate Mn_12_–stearate molecules at the surface. Nevertheless, we can clearly observe, that for the samples *SILS-COOMn*_12_*N*0, *SILSCOOMn*_12_*N*3, and *SILS-COOMn*_12_*N*6 SMMs are very densely distributed on the surface—molecule by molecule. No difference in distribution can be distinguished. In our previous work, we maintained that Mn_12_–stearate molecules were bonded at the surface in an umbrella-like configuration [15]. That is visible for the samples *SILS-COOMn*_12_*N*0 and *SILS-COOMn*_12_*N*3. Closer inspection of the TEM image of *SILS-COOMn*_12_*N*6 sample reveals one important detail, however. This sample shows Mn_12_–stearate molecules that are bounded in different orientations like those marked in the Figure 5.

This orientation of the SMMs can be observed for the samples *SILS-COOMn*_12_*N*9, *SILS-COOMn*_12_*N*12, and *SILS-COOMn*_12_*N*15 as well. Moreover, for these samples, we can clearly see a decrease in the number of anchored Mn_12_–stearate molecules. Why then can we observe a reduction in the anchored SMMs’ density after applying at least six spacers per anchoring unit? This finding seems to be inconsistent with the DPASV results. The explanation can be found by analyzing the molecular structure of the Mn_12_ molecule [22]. It has the form of a flattened ellipsoid with organic ligands located four each flat side and eight on the circumference of the molecule (a total of sixteen). The umbrella-like arrangement involves, probably, two to four bonds. In such a configuration the molecule is rigidly attached to the surface. When only one anchor is involved for immobilization of the SMMs, the molecule is relatively free, and arrangements different than umbrella-like are also possible. Looking at the TEM results we can say that the concentration of the spacer units of six per single anchoring group involves the single bond of the Mn_12_–stearate to the surface. Concentrations of the spacers of below this value do not cause an increase in the number of anchored SMMs but an increase the number of bonds between Mn_12_ and the support. This conclusion is in agreement with DPASV results: when the molecule is anchored via four bonds, its detection involves the exchange of four electrons and only one for a single bond. Thus, we observe continuous diminishing in the area under the DPASV, while TEM imaging shows roughly the same number of immobilized magnetic molecules, up to the six spacers per anchoring unit.

Here we must remark that some other research was carried out to check the chemical compositions of our specimens: energy-dispersive X-ray spectroscopy (EDX – FEI Tecnai G2 20 X-TWIN with an LaB6 emission source and an FEI Eagle 2 K CCD camera and X-Ray microanalyzer EDX—Thermo Fisher Scientific Inc., Waltham, MA, USA), Raman spectroscopy (confocal Raman microscope CRM alpha 300, WITec Inc., Ulm, Germany), and SQUID magnetometry (MPMS magnetometer, Quantum Design Inc., San Diego, CA, USA). Due to the reason of very low concentrations of the functional groups, EDX and Raman did not bring unambiguous results. The SQUID magnetometry proved that the Mn_12_–stearate molecules did not lose their SMMs properties. Moreover, SQUID research revealed extremely interesting behaviors of the material (a quantum tunneling phenomenon, non-linear dependence of the coercive field on the Mn_12_ concentration, and many more). The description of the magnetic properties of the materials is very extensive and is presented in a pending article. Magnetic research did not prove the light shed on the connection between Mn_12_–stearate and silica support though. For this reason, the only information about the chemical bonding between SMMs and the substrate was provided by DPASV.

Summing up both TEM and DPASV research, we observed that with the increase in the number of spacer units up to six per single anchoring molecule, we dealt with a decreasing of bonds between the support and Mn_12_–stearate, not diminishing the number of immobilized SMMs though. The reducing number of anchored magnetic molecules can be observed when the number of spacers is more than six per one anchoring molecule. Moreover, we can say that the samples *SILS-COOMn*_12_*N*6 or *FTO-COOMn*_12_*N*6 are optimal: they possess the largest possible numbers of immobilized SMMs with the smallest possible numbers of bonds. For the case of immobilization with Mn_12_ with more than a single anchoring point, the molecule is relatively rigid. Its mobility is limited. Therefore, the samples with six spacers contained the highest possible concentration of non-rigid, single-molecule magnets. This fact is reflected in the magnetic properties of such materials (work in preparation).

## Figures and Tables

**Figure 1:**
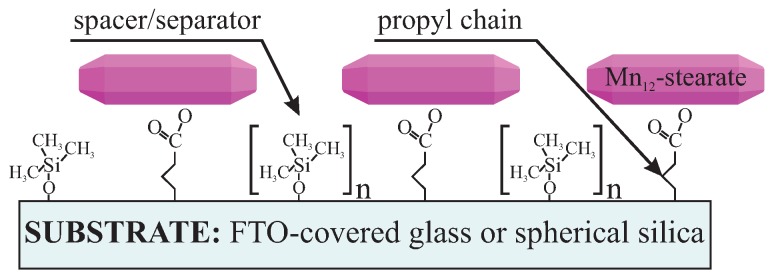
Schema of the proposed material: Mn_12_–stearate single-molecule magnets distributed onto FTO glass or spherical silica nanoparticles with assumed distributions. The intervals between Mn_12_–stearate single-molecule magnets (SMMs) can be controlled by tuning the numbers of the spacer groups (n) with respect to the number of the Mn_12_–stearate-containing units.

**Figure 2:**
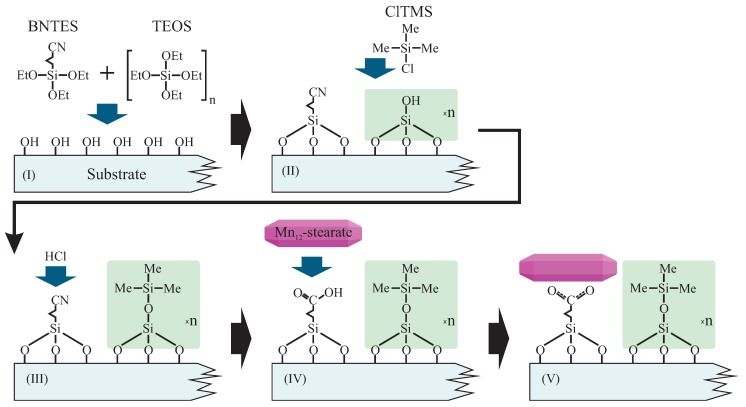
Schema of the functionalization route allowing for the statistical control of the distances between anchored Mn_12_–stearate single-molecule magnets. Abbreviations: BNTES—butyronitrile triethoxysilane, TEOS—tetraethyl orthosilicate, ClTMS—Chlorotrimethylsilane, Me—methyl groups, Et—ethyl units.

**Figure 3:**
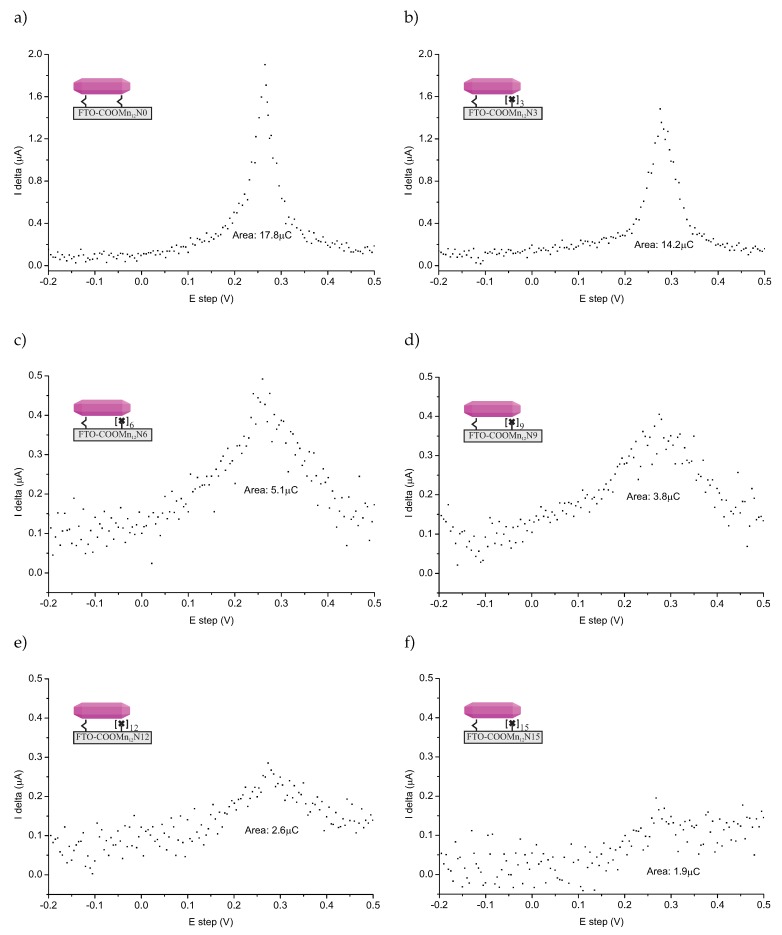
Differential pulse anodic stripping voltammetry (DPASV) recorded for FTO covered glass functionalized by Mn_12_-stearate molecules anchored via propyl-carbonic groups distributed onto the surface with various densities: full functionalization (no spacers between functionalities—sample *FTO-COOMn*_12_ *N0*—(**a**)), three (*FTO-COOMn*_12_ *N3*—(**b**)), six (*FTO-COOMn*_12_*g N6*—(**c**)), nine (*FTO-COOMn*_12_*g N9*—(**d**)), 12 (*FTO-COOMn*_12_*g N12*—(**e**)), and 15 (*FTO-COOMn*_12_*g N15*—(**f**)) spacers between anchoring units. Note, that the Y axis scale is larger for the cases of (**a**) and (**b**).

**Figure 4:**
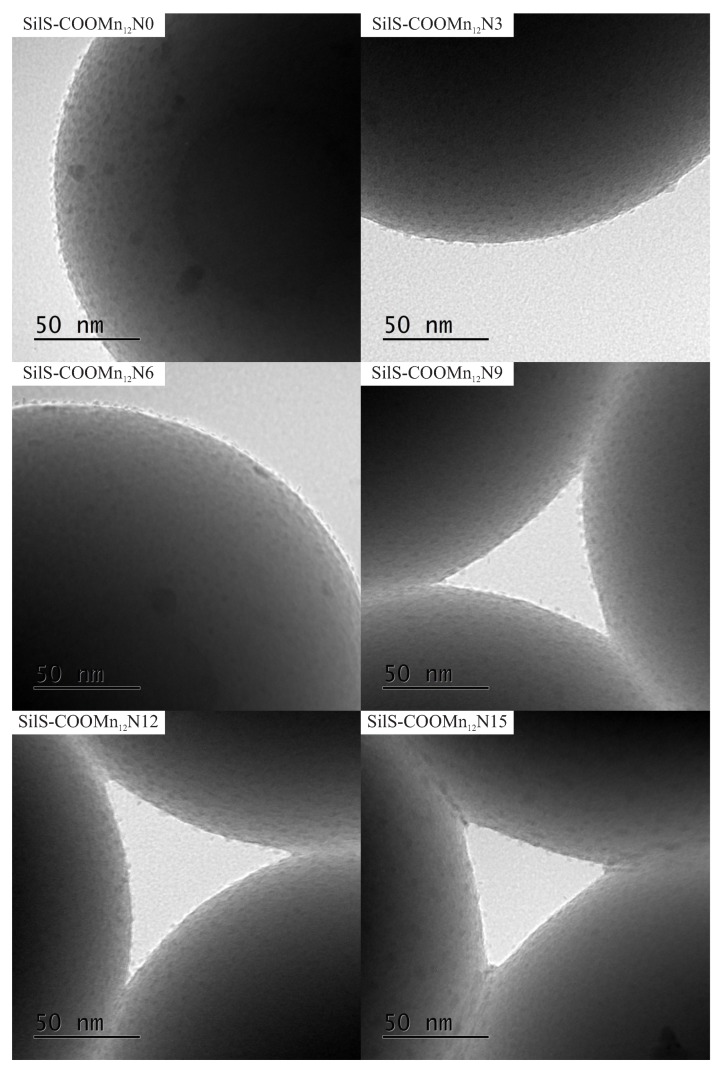
The transmission electron microscopy images of the spherical silica-based materials containing individual Mn_12_–stearate molecules attached to the surface with various concentration of the anchoring units.

**Figure 5:**
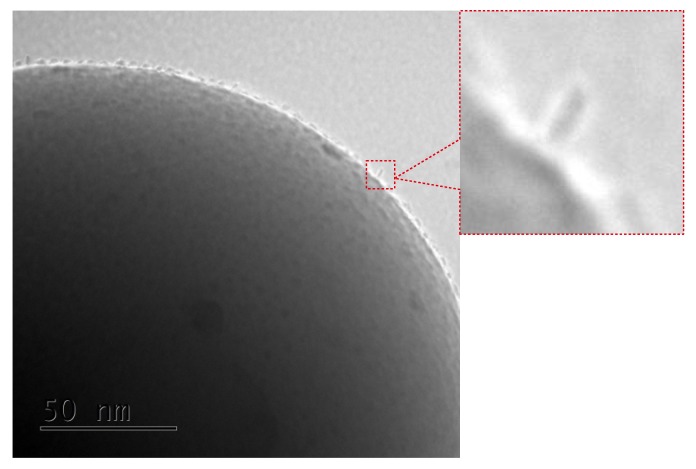
Closer inspection of the TEM image of the *SILS-COOMn*_12_*N*6 sample: enlarged part of the microphotograp shows the Mn_12_–stearate molecules attached in different configurations than the umbrella-like.

## Supplementary Materials

The following are available online at https://www.mdpi.com/2079-4991/9/12/1730/s1, Figure S1: The experiment verifying the role of anchoring units—the case of spherical silica: the transmission electron microscopy images of the materials at different stages of the synthesis and with the application of various steps of the procedure. Pure spherical silica (a), pure spherical silica functionalized by Mn_12_–stearate (b), silica with carbonic acid units and spacers in proportion 1:3 (c), silica with carbonic acid units and separators in ratio 1:3 functionalized by Mn12-st (d). Figure S2: The experiment verifying the role of anchoring units—the case of FTO-glasses: the DPASV results for the materials at different stages of the synthesis and with the application of various steps of the procedure. Pure spherical silica (a), pure spherical silica functionalized by Mn_12_–stearate (b), silica with carbonic acid units and spacers in proportion 1:3 (c), silica with carbonic acid units and separators in ratio 1:3 functionalized by Mn_12_–stearate (d). Figure S3: The DPASV results for of the spherical silica-based materials containing individual Mn_12_–stearate molecules attached to the surface with various concentration of the anchoring units (N means number of the spacer with regards to single anchoring group), juxtaposed with a pure FTO-glasse (FTO-pure), and FTO-glass functionalized by Mn12-stearate molecules (with no use of anchoring units). Figure S4: The transmission electron microscopy images of the spherical silica-based materials containing individual Mn_12_–stearate molecules attached to the surface with various concentration of the anchoring units (N means number of the spacer with regards to single anchoring group), juxtaposed with a pure spherical silica (SilS-pure), and pristine silica functionalized by Mn_12_–stearate molecules (with no use of anchoring units—SilS-Mn_12_).

## Abbreviations

The following abbreviations are used in this manuscript:

FTO	Fluoride doped Tin Oxide
Mn_12_ac_16_	[*Mn*_12_*O*_12_(*CH*_3_(*CH*_2_)_16_*CO*_2_)_11_(*CH*_3_*CO*_2_)_5_(*H*_2_*O*)_4_]·2*CH*_3_*COOH*
Mn_12_–stearate	derivative of Mn_12_ containing strearic acid ligands
