# Supplementary material for: How to Control the Distribution of Anchored, Mn12–Stearate, Single-Molecule Magnets
Source: Nanomaterials (Basel). 2019 Dec 4;9(12):1730. doi: 10.3390/nano9121730 (PMC6955952; doi:10.3390/nano9121730)
Supplement: Supplementary file 1 [file nanomaterials-09-01730-s001.pdf]

How to control the distribution of anchored Mn_{12}-stearate single-molecule magnets?

Supplementary Materials

Magdalena Laskowska, Oleksandr Pastukh, Dominika Kuźma, and Lukasz Laskowski

Additional experiments

Verifying the role of anchoring units

To additionally confirm that SMMs are chemically bonded at the silica surface via propyl-carbonic acid groups, as we assume, not only adsorbed, we carried out an additional experiment: functionalization of the pure substrates (with no anchoring units) by magnetic molecules. Samples in various stages of the syntheses were measured via transmission electron microscopy (spherical silica) and differential pulse anodic stripping voltammetry (DPASV). We juxtaposed results with the complete procedure, including grafting by the anchored groups. Obtained results can be seen in the fig. S1 for spherical silica and fig. S2 for FTO-glass. For both cases we chose the material containing three spacer units as representative.

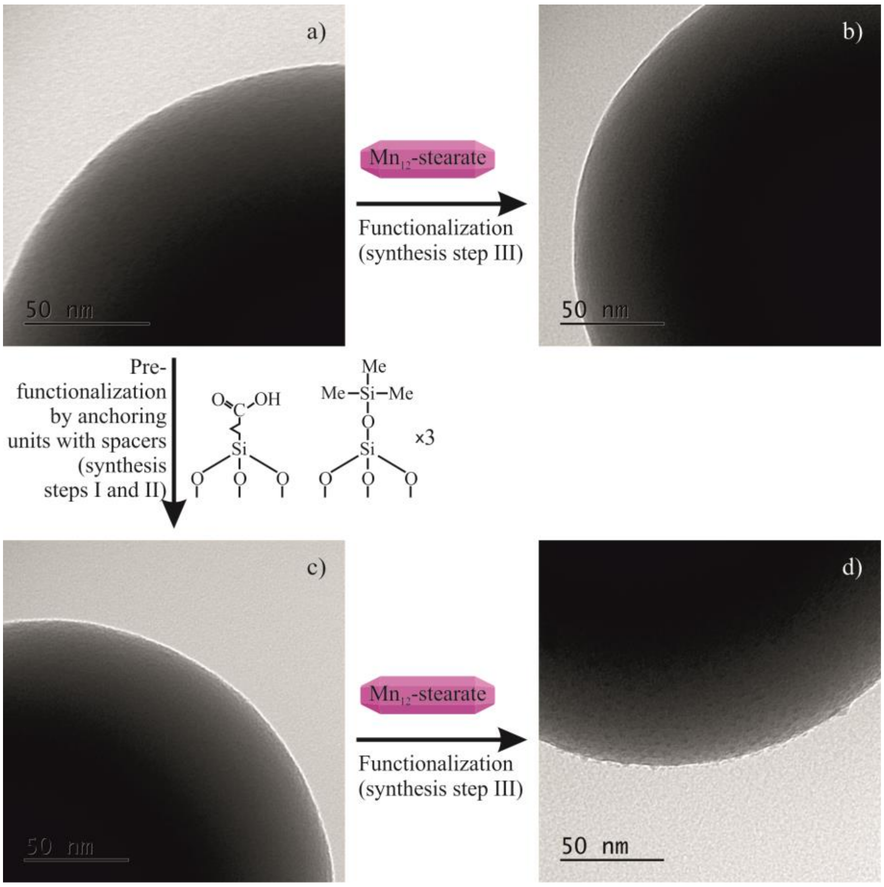

Fig. S1. The experiment verifying the role of anchoring units – the case of spherical silica: the transmission electron microscopy images of the materials at different stages of the synthesis and with the application of various steps of the procedure. Pure spherical silica (a), pure spherical silica functionalized by Mn_{12}- stearate (b), silica with carbonic acid units and spacers in proportion 1:3 (c), silica with carbonic acid units and separators in ratio 1:3 functionalized by Mn_{12}- stearate (d)

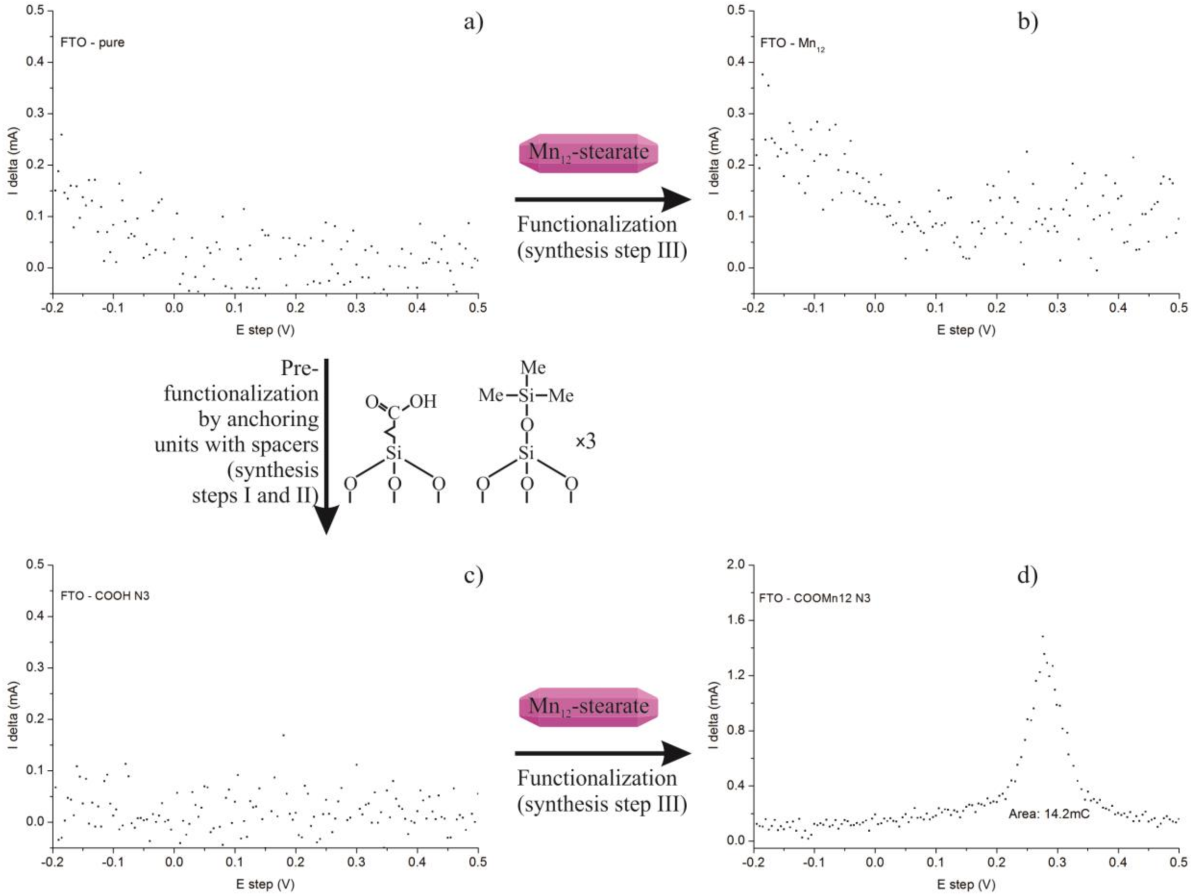

Fig. S2. The experiment verifying the role of anchoring units – the case of FTO-glasses: the DPASV results for the materials at different stages of the synthesis and with the application of various steps of the procedure. Pure spherical silica (a), pure spherical silica functionalized by Mn_{12}- stearate (b), silica with carbonic acid units and spacers in proportion 1:3 (c), silica with carbonic acid units and separators in ratio 1:3 functionalized by Mn_{12}-stearate (d)

As can be seen in fig. S1 b, resulting material did not contain any visible (under TEM microscope) molecules at the surface. On the contrary, the material possessing carbonic acid anchoring units was successfully functionalized: the surface seems to be covered by Mn_{12}-st molecules. Similarly for the case of FTO-covered glasses. We can clearly see that only for the case of pre-functionalization by carbonic acid units, some charge transport was detected by stripping volamperometry. On this basis, we can conclude that the carbonic acid groups bonded SMMs, and the Mn_{12}-st molecules are anchored at the silica surface via propyl-carbonic acid groups with a high probability.

Investigations of the pristine substrates and pre-functionalysed supports

We carried out the DPASV and TEM microscopy also for the pristine (non-functionalised) substrates and also for pre-functionalised by carbonic acid units and separators in various ratios. The DPASV results can be seen in fig. S3, while TEM images are presented in Fig. S4

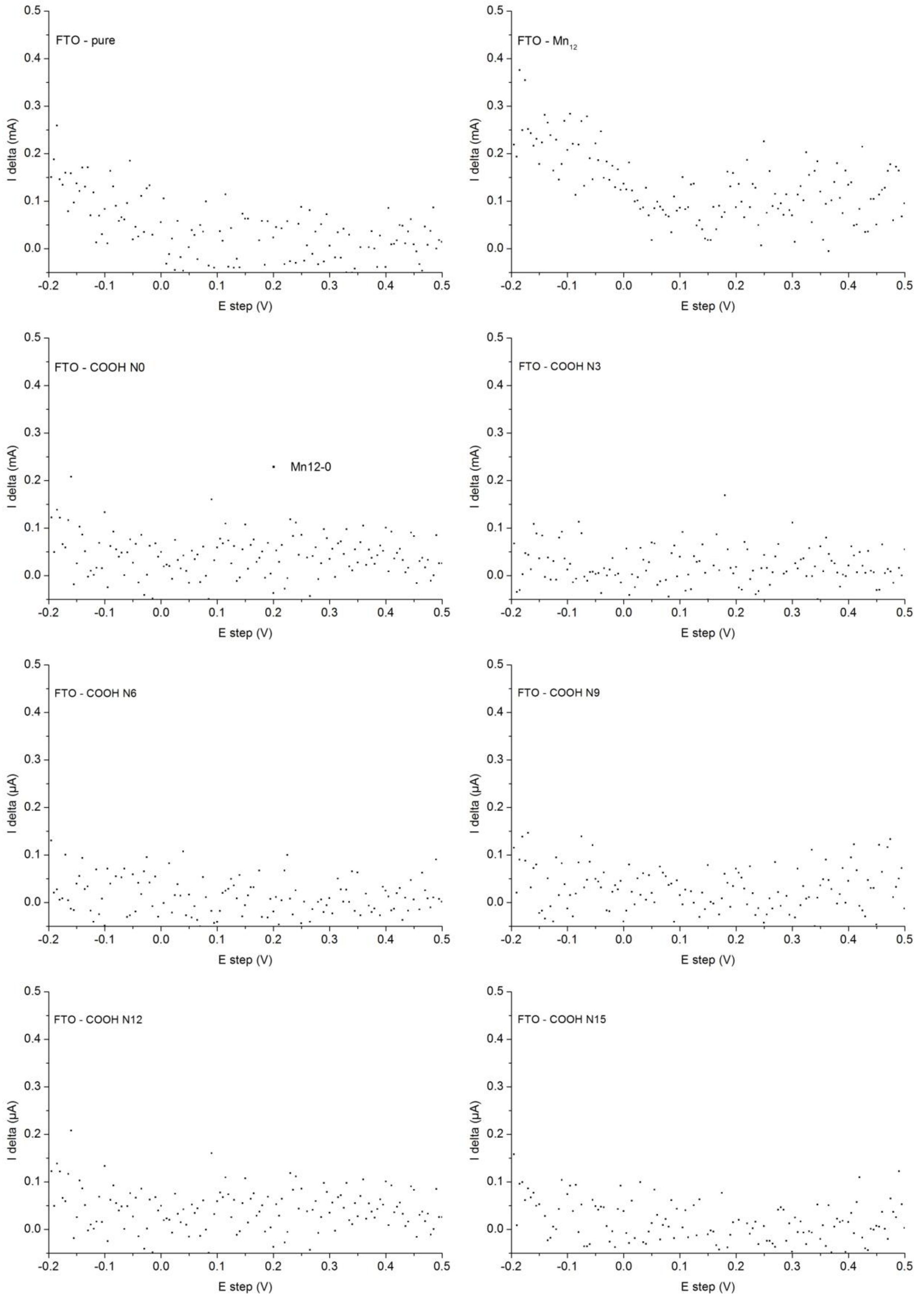

Fig. S3. The DPASV results for of the spherical silica-based materials containing individual Mn_{12}-stearate molecules attached to the surface with various concentration of the anchoring units (N means number of the spacer with regards to single anchoring group), juxtaposed with a pure FTO-glasse (FTO-pure), and FTO-glass functionalized by Mn_{12}-stearate molecules (with no use of anchoring units)

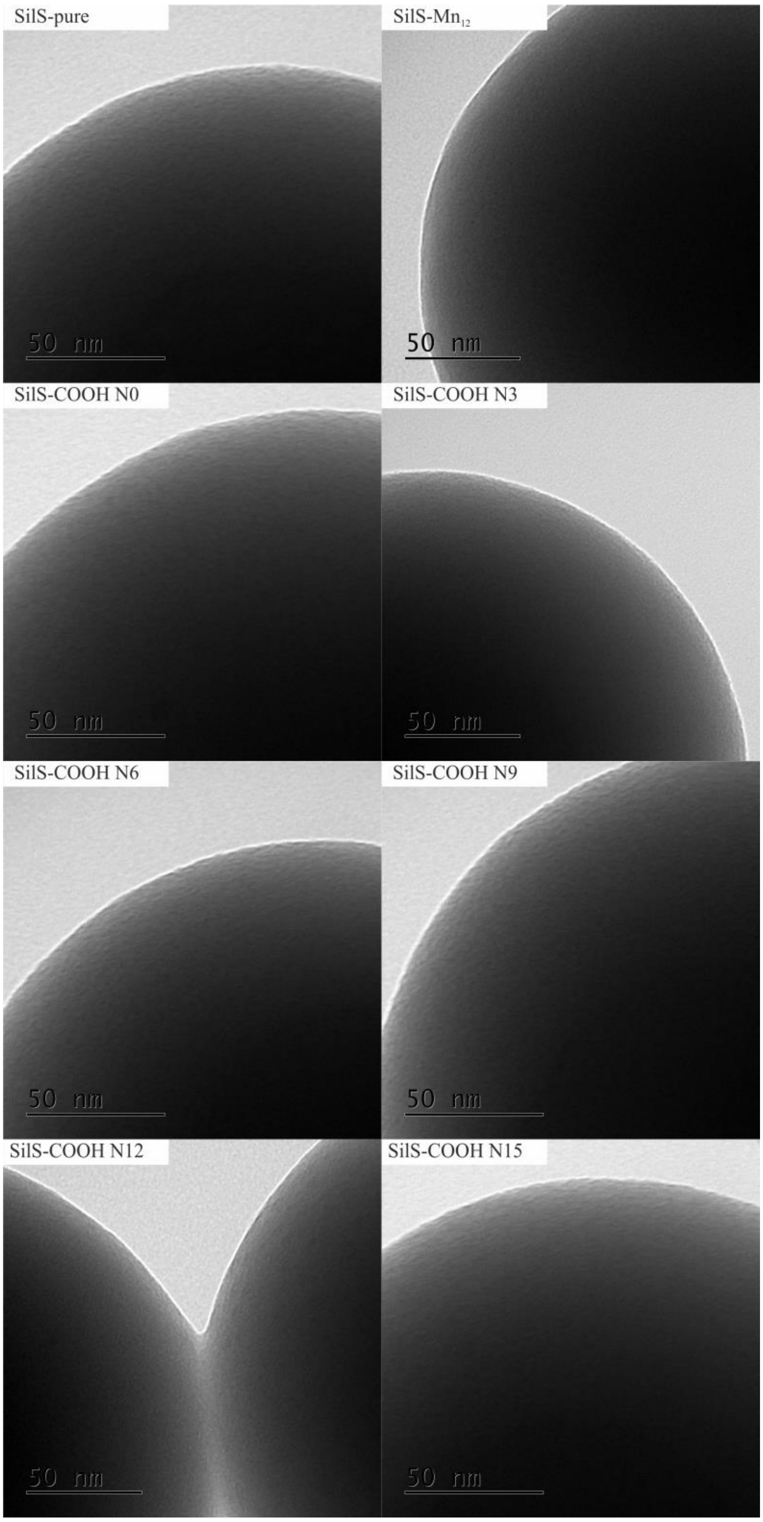

Fig. S4. The transmission electron microscopy images of the spherical silica-based materials containing individual Mn_{12}-stearate molecules attached to the surface with various concentration of the anchoring units (N means number of the spacer with regards to single anchoring group), juxtaposed with a pure spherical silica (SilS-pure), and pristine silica functionalized by Mn_{12}-stearate molecules (with no use of anchoring units – SilS-Mn_{12})

As it can be clearly seen, none of the reference samples shows any attached Mn_{12}-stearate molecules, that can be detected utilizing TEM or DPASV.
